# TEB vs. Swan-Ganz catheter: hemodynamic monitoring agreement in off-pump coronary artery bypass grafting

**DOI:** 10.1080/07853890.2026.2719250

**Published:** 2026-08-27

**Authors:** Weiling Wang, Xiangmei Bu, Quanyi Zhang, Feng Qi, Haiqing Gao

**Affiliations:** a Department of Geriatric Medicine, Laboratory of Gerontology and Anti-Aging Research Center, Qilu Hospital of Shandong University, Jinan, China; b Department of Anesthesiology, Shandong Medical and Pharmaceutical University Hospital, Binzhou, China; c Department of Anesthesiology, Qilu Hospital of Shandong University, Jinan, China

**Keywords:** Thoracic electrical bioimpedance (TEB), Swan-Ganz catheter, hemodynamics, off-pump coronary artery bypass grafting (OPCABG), perioperative monitoring

## Abstract

**Objective:**

To compare the agreement of hemodynamic parameters measured by Swan-Ganz catheter and thoracic electrical bioimpedance (TEB) in patients with coronary heart disease (CHD) undergoing coronary artery bypass grafting (CABG), and to evaluate the clinical application value of TEB in perioperative hemodynamic monitoring.

**Methods:**

A total of 42 CHD patients who underwent off-pump coronary artery bypass grafting (OPCABG) in the Department of Cardiac Surgery, Binzhou Medical University Hospital, from August 2021 to March 2022 were enrolled in this prospective observational study. All patients received continuous hemodynamic monitoring using both TEB and Swan-Ganz catheter throughout the surgical procedure, with simultaneous data collection. Cardiac output (CO), Stroke volume (SV), stroke volume index (SI), and end-diastolic volume (EDV) were recorded at two key time points (before thoracotomy and after chest closure) for statistical analysis.

**Results:**

Bland-Altman corrected for repeated measurements revealed mean bias of −0.30 L/min (CO), −9.10 mL (SV), −5.10 mL/m^2^ (SI), and 69.80 mL (EDV); corresponding 95% limits of agreement (LOA) were −2.50 to 1.80 L/min, −47.70 to 29.60 mL, −27.10 to 16.80 mL/m^2^, and −55.45 to 195.10 mL respectively. The 95% LOA of all four indices fell within predefined clinical acceptable thresholds, whereas paired t-test demonstrated statistically significant inter-method differences for all measured parameters.

**Conclusion:**

TEB serves as a reliable, non-invasive tool for real-time continuous trend monitoring of CO, SV, SI, and EDV in critically ill surgical patients, especially those for whom invasive monitoring is contraindicated. This study supports the clinical application of TEB as an alternative or supplementary method for perioperative hemodynamic assessment in OPCABG patients.

## 1. Introduction

With the accelerating global population aging, the incidence of cardiovascular diseases (CVDs) has been on a steady rise. Coronary heart disease (CHD), as a major subtype of CVDs, poses a severe threat to public health worldwide. In 2019, approximately 197 million people were affected by CHD globally, with 9.14 million deaths attributed to this disease; China ranks among the countries with the highest CHD-related mortality [1,2]. According to the Report on Cardiovascular Health and Diseases in China, the number of CHD patients in China had reached around 11.39 million by 2021, with an annually increasing incidence rate. Coronary artery bypass grafting (CABG) remains the most widely adopted definitive treatment for severe CHD complicated by multi-vessel diffuse coronary artery lesions [3]. Compared with pharmacotherapy alone, CABG confers more favorable prognostic outcomes for eligible patients [4].

During CABG, especially off-pump coronary artery bypass grafting (OPCABG), surgical manipulation often requires cardiac displacement to expose target vessels [5]. Alterations in the heart’s normal anatomical position, along with mechanical stretching and compression, can induce significant fluctuations in cardiac output (CO) and other hemodynamic parameters [6]. Such perturbations may predispose patients to serious adverse events, including myocardial ischemia, hypotension, and malignant arrhythmias [7–9]. Therefore, accurate, real-time monitoring of hemodynamic indices is crucial for ensuring the smooth progression of surgery and safeguarding patient safety.

Methods for CO measurement are generally classified into invasive and non-invasive modalities. The invasive approach, typified by the Swan-Ganz catheter (thermodilution technique), is traditionally regarded as the clinical gold standard due to its high precision [10]. Beyond monitoring pulmonary artery pressure (PAP), the Swan-Ganz catheter enables real-time assessment of cardiac function and early detection of myocardial ischemia, making it a cornerstone of hemodynamic monitoring in intensive care units (ICUs) [11]. Additionally, derived parameters such as end-diastolic volume (EDV), stroke volume (SV), and stroke volume index (SI) can be calculated by integrating heart rate (HR) and body surface area (BSA) data [12]. However, this invasive procedure is technically complex, requiring proficient medical personnel, and is associated with non-negligible risks, including arrhythmias, pulmonary infarction, vascular or cardiac rupture, balloon rupture, and catheter entanglement [13].

In contrast, non-invasive techniques – such as echocardiography, cardiac nuclear imaging, magnetic resonance imaging (MRI), and thoracic electrical bioimpedance (TEB) – offer advantages of simplicity, safety, cost-effectiveness, and rapid data acquisition [14–17]. Among these, TEB has garnered particular favor among clinicians due to its ability to quantify changes in thoracic impedance, thereby reflecting intrathoracic blood flow dynamics and cardiac function [18]. TEB has been extensively applied in clinical cardiovascular assessments, cardiac performance evaluation, and postoperative monitoring of cardiac patients, with promising preliminary results [19].

Owing to its non-invasive nature and proven reliability, TEB has been increasingly adopted in ICUs and coronary care units (CCUs). However, its application in surgical settings – especially during OPCABG – has raised concerns regarding measurement agreement, as electrosurgical devices and intraoperative monitors may interfere with impedance signals. Existing evidence on the agreement between TEB-derived and thermodilution-derived CO values remains contradictory. For instance, one study reported high agreement between the two methods, while a 2010 meta-analysis involving over 400 patients demonstrated an overall percentage error of 42.9% for TEB-measured CO (exceeding the clinically acceptable threshold of 30%), indicating poor agreement with thermodilution-derived CO [20].

Given the clinical demand for reliable intraoperative hemodynamic monitoring and the conflicting evidence on TEB’s performance in OPCABG settings, this study aimed to: (1) compare the agreement of key hemodynamic parameters (CO, SV, SI, EDV) measured by TEB and the Swan-Ganz catheter during OPCABG; and (2) evaluate the clinical feasibility and agreement performance of TEB for intraoperative hemodynamic monitoring in CHD patients undergoing OPCABG.

## 2. Study design

### 2.1. Participants

This prospective observational study was conducted at the Department of Cardiac Surgery, Binzhou Medical University Hospital, between August 2021 and March 2022. Eligible participants were patients diagnosed with CHD who underwent OPCABG and required intraoperative hemodynamic monitoring using both TEB and Swan-Ganz catheterization.

Baseline categorical variables were clearly defined in this study. Smoking history was dichotomized into non-smoker and smoker (passive smoking was not counted). Metformin medication was set as categorical variable with fixed daily dosage of 1500 mg (0.5 g three times daily oral administration for patients receiving metformin).

It should be noted that the count of diseased coronary vessels was only used for baseline demographic matching of enrolled patients, and this indicator could not reflect the overall severity and stenosis degree of coronary atherosclerosis.

Exclusion criteria were defined as follows: (1) contraindications to Swan-Ganz catheter puncture (e.g. coagulation disorders, severe pulmonary hypertension) or failed catheter insertion; (2) pre-existing malignant arrhythmias or severe valvular heart disease; (3) severe thoracic deformities that might interfere with TEB electrode placement or signal acquisition; (4) inability to obtain written informed consent from patients or their legal representatives.

This clinical research study was prospectively designed. The complete study protocol, research questionnaires and written informed consent documents were collectively reviewed and formally approved by the Ethics Committee of Qilu Hospital of Shandong University (Approval Date: October 18, 2018; Approval No.: KYLL-2018-376). Prior to participant enrollment, written informed consent was obtained from each subject or their legal guardians voluntarily. All clinical experimental procedures involved in this research were strictly conducted in compliance with the principles outlined in the Declaration of Helsinki (2013 revision).

This prospective observational study strictly followed the Strengthening the Reporting of Observational Studies in Epidemiology (STROBE) reporting guidelines. Consecutive eligible patients who met the inclusion criteria during the study period were enrolled, and no eligible patients were excluded for non-medical reasons.

### 2.2. Instruments

1. **TEB Monitoring System**
  HEMO-ZH01 non-invasive hemodynamic monitor (Shenzhen Zehui Medical Technology Co., Ltd., Shenzhen, China), equipped with dedicated disposable electrodes for thoracic impedance measurement.
2. **Swan-Ganz Catheterization System**

HemoSphere Patient Monitor, HemoSphere Swan-Ganz module, and corresponding accessories (Edwards Lifesciences LLC, Irvine, CA, USA), including 7.5-Fr balloon-tipped thermodilution catheters and pressure transduction kits.

### 2.3. Procedures

All patients underwent standardized general anesthesia and intraoperative monitoring. Routine hemodynamic monitoring included electrocardiography (ECG), pulse oximetry (SpO_2_), non-invasive blood pressure (NIBP), and direct arterial pressure (*via* radial or femoral artery cannulation).

Anesthesia induction was performed with midazolam (5 μg/kg), sufentanil (8–20 μg/kg), vecuronium (0.08–0.1 mg/kg) or cisatracurium (0.15–0.2 mg/kg), and etomidate (0.3–0.5 mg/kg). After tracheal intubation, a transesophageal echocardiography (TEE) probe was placed to monitor myocardial motion and exclude intraoperative myocardial ischemia. Anesthesia maintenance was achieved with sevoflurane (1–2 vol%) inhalation combined with continuous intravenous infusion of propofol (4–12 mg·kg^−1^·h^−1^) and remifentanil (0.1–0.3 μg·kg^−1^·min^−1^).

Following successful anesthesia induction, two trained cardiac anesthesiologists simultaneously performed TEB and Swan-Ganz catheter monitoring. Hemodynamic parameters, including CO, SV, SI, and EDV, were recorded at two predefined time points: 1) before thoracotomy (T1, baseline status after anesthesia induction and stabilization); 2) after chest closure (T2, post-surgical stabilization before anesthesia emergence).

1. **Swan-Ganz Catheter Monitoring**
  The HemoSphere Patient Monitor was calibrated and initialized according to the manufacturer’s instructions, and demographic data (age, gender, height, weight, body surface area) were entered into the system. Under ultrasound guidance (Philips EPIQ 5, Philips Healthcare, Amsterdam, the Netherlands), an introducer sheath was placed into the right internal jugular vein. The distal lumen of the Swan-Ganz catheter was connected to a pressure transducer (zero-calibrated to atmospheric pressure at the midaxillary line), and the thermistor connector was attached to the HemoSphere module. A 1.5 mL inflation syringe was connected to the balloon port.
  The catheter was advanced through the sheath to 15 cm under fluoroscopic guidance, after which the balloon was fully inflated with air (1.0–1.5 mL). The catheter was then advanced incrementally while continuous pressure waveform monitoring was performed to confirm positioning: right atrium → right ventricle → pulmonary artery → pulmonary artery wedge pressure (PAWP) position. Once the catheter tip was stabilized in the pulmonary artery (confirmed by characteristic pressure waveforms), the balloon was deflated, and the catheter was secured with sutures. Hemodynamic parameters were automatically recorded by the HemoSphere monitor at each time point, with three consecutive measurements averaged for analysis.
2. **TEB Monitoring**

To avoid signal interference between respiratory monitoring and TEB, the respiratory impedance function of the routine ECG monitor was disabled. Patients were maintained in a supine position with minimal movement throughout monitoring. The skin over the electrode placement sites (anterior chest wall, midline sternum, and bilateral mid-axillary lines) was cleaned with alcohol swabs to remove grease and debris, ensuring optimal electrode-skin contact. Disposable TEB electrodes were applied according to the manufacturer’s standard protocol (four electrodes placed in a tetrapolar configuration: two at the base of the neck, two at the level of the xiphoid process).

After entering patient demographic data into the CSM3000 monitor, the device was activated to record impedance ECG, impedance cardiogram (ICG), and cardiac impedance differential curves. Monitoring was continued for 5 min to allow signal stabilization. Once stable waveforms were obtained, data were frozen and stored three times at 30-second intervals. The mean values of CO, SV, SI, and EDV from the three recordings were used for subsequent analysis.

### 2.4. Statistical analysis

All statistical analyses were performed using MedCalc Statistical Software version 20.011 (MedCalc Software Ltd., Ostend, Belgium). Normality of continuous data was tested using the Shapiro-Wilk test. Normally distributed measurement data are expressed as mean ± standard deviation (SD) (x̅ ± s). Paired sample t-tests were used to compare inter-method mean differences of hemodynamic parameters at each time point; notably, paired t-test only assesses whether the average difference between two measurement approaches differs statistically from zero and cannot be used to judge measurement agreement. Agreement between methods was exclusively evaluated *via* repeated-measures corrected Bland-Altman analysis incorporating mean bias, 95% limits of agreement (LOA), and predefined clinical acceptable thresholds. Bland-Altman analysis was performed to assess the agreement between the two monitoring methods, with 95% LOA calculated. A two-tailed P-value < 0.05 was considered statistically significant. All statistical analytical methods adopted in this study were consistent with standard methodological specifications for prospective observational clinical studies, fully complying with STROBE statement requirements for data processing, repeated-measures analysis, and agreement evaluation reporting.

This study was designed as a prospective exploratory clinical investigation, and no formal a priori sample size calculation was performed for Bland-Altman agreement analysis. The sample size of 42 patients was determined based on the available clinical cases meeting the strict inclusion and exclusion criteria during the study period and the common sample scale of published perioperative hemodynamic method-comparison studies. To quantify the precision of agreement estimates, 95% confidence intervals (95% CIs) for mean bias and 95% LOA were additionally calculated according to standard Bland-Altman statistical specifications.

In addition to paired t-test and Bland-Altman agreement analysis, we calculated the paired mean differences, 95% confidence intervals (95% CIs), and exact two-tailed P values for all hemodynamic parameters (CO, SV, SI, EDV) between TEB and Swan-Ganz measurements at T1 and T2 time points, respectively. The paired mean difference was defined as TEB value minus Swan-Ganz value. The 95% CIs of mean differences were computed based on student’s t-distribution. All exact P values were derived from paired sample t-tests for each time point to provide precise statistical comparison results.

In this study, each patient generated paired measurements at two independent intraoperative time points (T1 and T2), with three replicate readings averaged at each time point to eliminate random instrument measurement error. All final analytical data were time-point-specific paired mean values. Considering the repeated measures design, we adopted a structured statistical strategy to avoid biased uncertainty estimation: (1) The paired t-test was performed separately for each time point to eliminate intra-individual correlation interference; (2) A repeated-measures corrected Bland-Altman model was applied for agreement analysis, which accounts for within-subject variability and non-independent repeated observations from the same patient. Unlike the conventional simple Bland-Altman approach that assumes independent data, this corrected method calculates bias and 95% LOA based on residual errors after adjusting for individual patient clustering effects, thereby avoiding underestimation of measurement uncertainty.

For Bland-Altman agreement analysis, a priori clinically acceptable LOA for each hemodynamic parameter were strictly defined based on published perioperative cardiac monitoring consensus and clinical expert criteria, to enable objective clinical judgment of measurement agreement. The predefined clinically acceptable difference thresholds were set as follows: CO: ±2.0 L/min, SV: ±50 mL, SI: ±30 mL/m^2^, and EDV: ±200 mL. These thresholds represent the maximum measurement discrepancies that do not affect perioperative hemodynamic assessment, fluid resuscitation strategy, and clinical decision-making in OPCABG patients. Agreement between the two methods was defined as the calculated 95% LOA fully falling within the predefined clinical acceptable range.

## 3. Results

### 3.1. Demographic and baseline characteristics

A total of 43 patients with coronary artery disease scheduled for OPCABG were initially enrolled in this study. One patient was excluded due to intraoperative hemodynamic instability that necessitated conversion to on-pump CABG. Finally, 42 patients were included in the final statistical analysis, consisting of 30 males and 12 females, with an age range of 48 to 77 years (mean ± SD, 65.08 ± 7.73 years). The mean body mass index (BMI) of the cohort was 24.04 ± 2.64 kg/m^2^. Detailed patient enrollment, screening, and exclusion processes were strictly documented following STROBE guidelines, with clear recording of the total initially enrolled population, excluded cases, and final analytical sample size. The baseline clinical characteristics of the enrolled patients are summarized in Table 1.

**Table 1.** General clinical characteristics of the included patients (*n* = 42).

Characteristic	Data
Gender, n (%)	
Male	30 (71.43)
Female	12 (28.57)
Age, Years, Mean ± SD (range)	65.08 ± 7.73 (48 ∼ 77)
Body Mass Index, kg/m², Mean ± SD	24.04 ± 2.64
Number of Stenotic Coronary Vessels (Baseline Matching Index), n (%)	
Single-Vessel Disease	23 (54.76)
Double-Vessel Disease	10 (23.81)
Triple-Vessel Disease	4 (9.52)
Other unclassified coronary lesions	5 (11.90)
Hypertension, n (%)	30 (71.43)
Diabetes Mellitus, n (%)	14 (33.33)
Cerebral Infarction, n (%)	7 (16.67)
Atrial Fibrillation, n (%)	2 (4.76)
Wolff-Parkinson-White Syndrome, n (%)	1 (2.38)
Smoking status (passive smoking excluded), n (%)	19 (45.24)
History of Alcohol Consumption, n (%)	9 (21.42)
Antihypertensive Medications, n (%)	
ACEI/ARB	16 (38.10)
CCB	9 (21.43)
Diuretics	5 (11.90)
Antidiabetic Medications, n (%)	
Thiazolidinediones	2 (4.76)
Metformin (fixed dose: 1500 mg/d, 0.5 g oral tid), n (%)	11 (26.19)
α‑Glucosidase Inhibitors	2 (4.76)
Insulin Secretagogues	3 (7.14)
Chinese Patent Medicine	1 (2.38)

**Abbreviations:** ACEI, angiotensin-converting enzyme inhibitors; ARB, angiotensin II receptor blocker; CCB, calcium channel blocker; SD, standard deviation.

### 3.2. Bland-Altman analysis of agreement between Swan-Ganz and TEB measurements

Bland-Altman analysis was performed to assess the agreement of key hemodynamic parameters measured by the Swan-Ganz catheter and TEB, and the results revealed minimal maximum absolute differences for all detected indices, indicating negligible systematic bias between the two monitoring methods. The mean bias (± SD) of CO was −0.30 ± 1.08 L/min, EDV 69.8 ± 63.91 mL, SV −9.10 ± 19.74 mL, and SI −5.10 ± 11.20 mL/m^2^. The 95% CIs for mean bias and 95% LOA were calculated to reflect the precision of measurement agreement estimates. Specifically, the 95% CI of mean bias was −0.59 to −0.11 L/min for CO, −13.40 to −4.72 mL for SV, −7.57 to −2.65 mL/m^2^ for SI, and 55.00 to 84.62 mL for EDV. Correspondingly, the 95% CIs for the lower and upper limits of agreement were (−2.88, −2.06) L/min and (1.37, 2.19) L/min for CO, (−55.20, −40.31) mL and (22.19, 37.08) mL for SV, (−31.29, −22.84) mL/m^2^ and (12.62, 21.07) mL/m^2^ for SI, as well as (−80.87, −30.03) mL and (169.66, 220.49) mL for EDV, respectively.

All agreement indicators were derived from the repeated-measures corrected Bland-Altman analysis with adjustment for intra-patient correlation of dual time-point observations. The corrected 95% LOA fully incorporated within-subject repeated measurement variability, ensuring rigorous estimation of measurement uncertainty and eliminating the statistical bias caused by non-independent data. Notably, the 95% LOA of all four parameters calculated in this study were further compared with the pre-defined clinically acceptable difference thresholds. Specifically, the 95% LOA of CO (−2.5 ∼ 1.8 L/min) was within the acceptable range of ±2.0 L/min; the 95% LOA of SV (−47.7 ∼ 29.6 mL) was within the acceptable range of ±50 mL; the 95% LOA of SI (−27.1 ∼ 16.8 mL/m^2^) was within the acceptable range of ±30 mL/m^2^; and the 95% LOA of EDV (−55.45 ∼ 195.1 mL) was within the acceptable range of ±200 mL. For all parameters, less than 5% of measurement points fell outside the 95% LOA.

Statistically significant inter-method mean differences were observed for all indices according to paired t-test results (Table 2), which reflected systematic numerical deviation between TEB and Swan-Ganz rather than disagreement; CO, SV and SI presented favorable absolute agreement within predefined clinical thresholds and could be interchangeably used for absolute numerical measurement in clinical practice; by contrast, EDV displayed prominent positive mean bias (69.8 mL) and wide 95% LOA spanning −55.45 ∼ 195.1 mL, indicating substantial discrepancy in absolute EDV values between two methods and insufficient agreement to support direct numerical interchange. Nevertheless, EDV’s overall LOA still fell within pre-set clinical cutoff, and consistent changing trends across perioperative timepoints were observed, enabling TEB to reliably track dynamic volume fluctuation rather than accurate absolute EDV quantification. Collectively, TEB achieves acceptable absolute agreement for CO, SV, SI and valid trend monitoring for EDV.

**Table 2.** Comparative analysis of hemodynamic parameters measured by Swan-Ganz and TEB at T1 and T2 time points (*n* = 42).

Parameters	Time point	Swan-Ganz (Mean ± SD)	TEB (Mean ± SD)	Paired mean difference (TEB-Swan-Ganz)	95% CI of difference	Exact P value
CO (L/min)	T1	4.25 ± 1.34	4.83 ± 1.37	0.58	(0.19∼0.96)	0.0043
	T2	4.87 ± 1.44	5.04 ± 1.41	0.17	(−0.15∼0.5)	0.2829
SV (mL)	T1	65.10 ± 18.89	78.55 ± 25.66	13.45	(6.99∼19.91)	0.0001
	T2	70.17 ± 17.87	77.17 ± 18.10	7	(1.02∼12.98)	0.0233
SI (mL/m²)	T1	37.62 ± 10.33	45.26 ± 14.07	7.64	(3.85∼11.44)	0.0002
	T2	40.33 ± 8.88	44.03 ± 9.25	3.7	(0.62∼6.78)	0.0203
EDV (mL)	T1	217.16 ± 56.81	137.83 ± 61.56	−74.16	(−102.11∼-46.21)	0
	T2	201.04 ± 41.95	128.14 ± 25.87	−77.45	(−94.38∼-60.52)	0

**Abbreviations:** CO, cardiac output; EDV, end-diastolic volume; SI, stroke volume index; SV, stroke volume; TEB, thoracic electrical bioimpedance; SD, standard deviation; CI, confidence interval.

**Figure 1.**
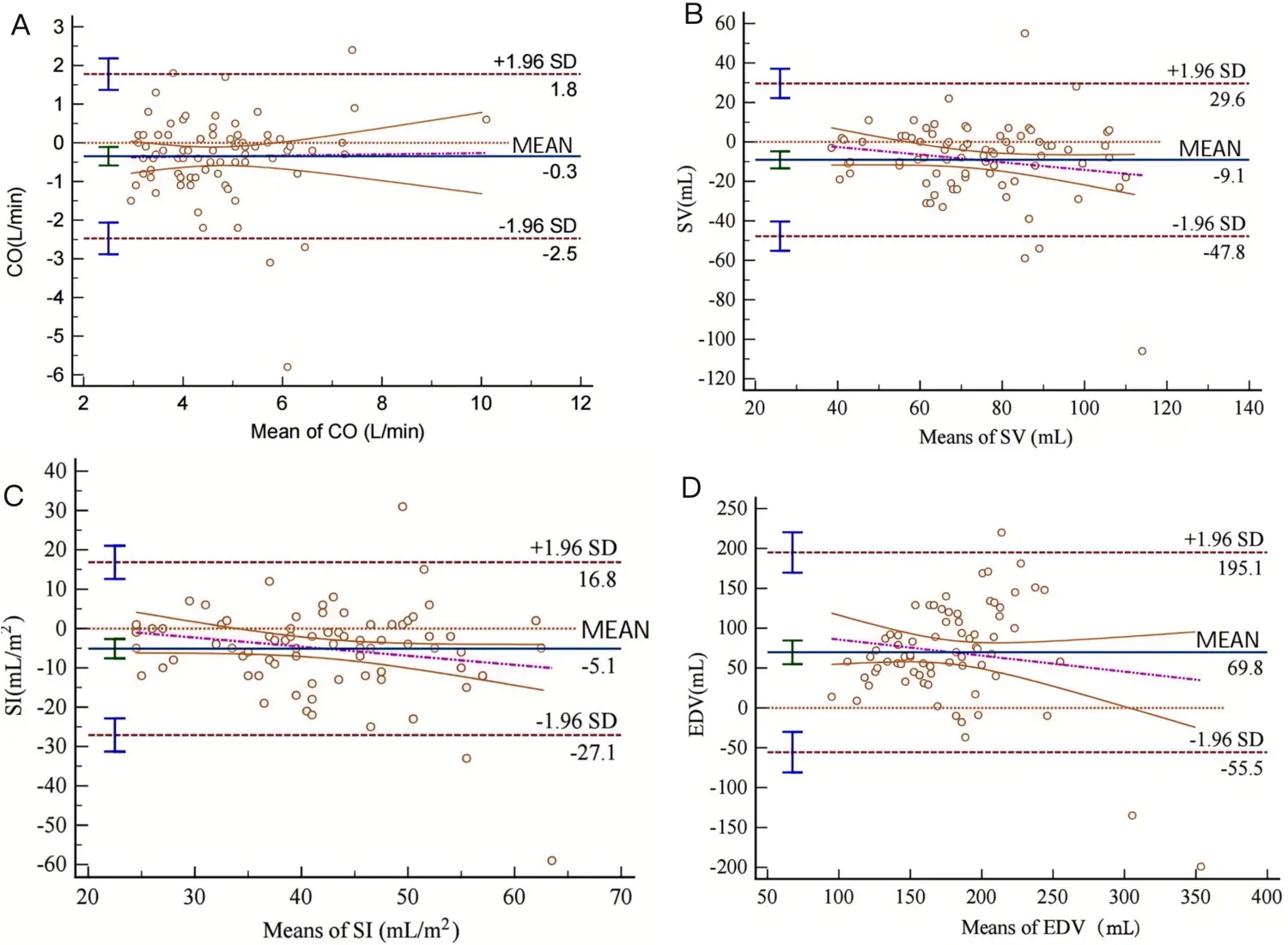
Results of Bland-Altman Analysis for Comparing Swan-Ganz and TEB Measurements. Solid horizontal lines denote mean bias, while dashed horizontal lines represent 95% limits of agreement (LOA). (A) CO (cardiac output, L/min); (B) SV (stroke volume, mL); (C) SI (stroke volume index, mL/m^2^); (D) EDV (end-diastolic volume, mL). Less than 5% of paired data points lie outside the 95% LOA for all four hemodynamic parameters.

To further intuitively present the raw comparative data between the two monitoring methods and clarify the clinical relevance of measurement differences, all paired quantitative results at T1 (before thoracotomy) and T2 (after chest closure) were summarized in Table 2, including the mean ± SD values of each parameter measured by Swan-Ganz and TEB, paired mean differences, 95% CIs of differences, and exact P values. Detailed quantitative results of the Bland-Altman analysis are presented in Table 3, and the corresponding Bland-Altman plots are shown in Figure 1.

**Table 3.** Bland-Altman Analysis results for hemodynamic parameters measured by Swan-Ganz and TEB.

Variable	Mean Difference	95% CI of Mean Bias	SD of Difference	Lower LOA Limit	95% CI for Lower LOA Limit	Upper LOA Limit	95% CI for Upper LOA Limit	Percentage of Points Outside LOA
CO (L/min)	−0.3	−0.59 to −0.11	1.08	−2.5	(−2.88, −2.06)	1.8	(1.37, 2.19)	4.90%
SV (mL)	−9.1	−13.40 to −4.72	19.74	−47.7	(−55.20, −40.31)	29.6	(22.19, 37.08)	4.90%
SI (mL/m²)	−5.1	−7.57 to −2.65	11.2	−27.1	(−31.29, −22.84)	16.8	(12.62, 21.07)	3.70%
EDV (mL)	69.8	55.00 to 84.62	63.91	−55.45	(−80.87, −30.03)	195.1	(169.66, 220.49)	4.10%

**Abbreviations:** CO, cardiac output; EDV, end-diastolic volume; LOA, limits of agreement; SI, stroke volume index; SV, stroke volume; TEB, thoracic electrical bioimpedance; SD, standard deviation; CI, confidence interval.

## 4. Discussion

Maintenance of a delicate balance between myocardial oxygen supply and demand is pivotal during the perioperative period of OPCABG, and real-time, accurate anesthetic and hemodynamic monitoring is the cornerstone of achieving this balance [21]. The Swan-Ganz catheter is the clinical gold standard for invasive hemodynamic monitoring, enabling continuous measurement of CO, EDV, pulmonary artery pressure, and dynamic assessment of right ventricular function [22]. In the present study, all enrolled patients underwent Swan-Ganz catheter monitoring during OPCABG without procedure-related adverse events, and the reliability of its derived hemodynamic parameters has been well established in clinical practice. TEB, a non-invasive hemodynamic monitoring technique, can simultaneously measure CO, SV, SI, and EDV – the same key indices as the Swan-Ganz catheter. This study was designed to compare the agreement of these hemodynamic parameters measured by the two methods, thereby evaluating the clinical feasibility and application value of TEB for intraoperative monitoring in OPCABG patients.

Perioperative fluid balance disturbance is a common clinical challenge in OPCABG patients, driven by preoperative fasting, anesthesia-induced vasodilation, and intraoperative blood loss [23]. Inadequate fluid resuscitation can lead to decreased CO and hypoperfusion of vital organs, while excessive fluid administration may cause cardiac overdistension, increased myocardial wall tension, elevated myocardial oxygen consumption, and reduced coronary blood flow – all of which can exacerbate myocardial ischemia [24]. Central venous pressure (CVP) monitoring and echocardiography are the most commonly used clinical methods for assessing volume status [25,26]. CVP monitoring reflects volume status *via* pressure measurements but is susceptible to interference from cardiac function, valvular heart disease, and thoracic pressure, leading to limited reliability in specific patient populations [27]. In contrast, TEB assesses SV by detecting cyclic changes in thoracic impedance caused by cardiac contraction and relaxation [28]; when combined with patient demographic data (height, weight) and vital signs (blood pressure, heart rate), it can non-invasively and continuously display CO, EDV, and other hemodynamic indices on a dedicated monitor, providing a practical approach for real-time perioperative volume status assessment [29].

In this study, the mean bias of EDV measured by the Swan-Ganz catheter and TEB was 69.8 mL, indicating a numerical difference between the two methods, which is likely attributable to their fundamentally distinct measurement theoretical frameworks and technical principles. Despite this quantitative discrepancy, the dynamic trends of EDV measured by the two methods were consistent before thoracotomy and after chest closure in all patients. This finding indicates that TEB can reliably reflect real-time changes in hemodynamic parameters and serve as a valid supplementary trend-monitoring tool during standardized OPCABG procedures in intraoperatively stable patients.

TEE is another widely used clinical tool for perioperative volume and cardiac function assessment, enabling real-time visualization of cardiac structure and motion, and early identification of myocardial ischemia-induced abnormalities in myocardial motion morphology – an essential assessment for determining surgical success in OPCABG patients [30]. All patients in this study underwent routine TEE monitoring, but the placement of a TEE probe may alter thoracic anatomical structure and thus interfere with thoracic impedance signals, potentially introducing bias into TEB measurements; this may be an additional factor contributing to the numerical differences between TEB and Swan-Ganz catheter-derived parameters. To minimize this interference, TEB data were collected under standardized TEE monitoring conditions for all patients, ensuring the internal validity of the study’s comparative analysis. While TEE can accurately measure CO, ejection fraction, and SV, its clinical application is limited by the need for specialized operator proficiency and its semi-quantitative nature for continuous dynamic monitoring – limitations that TEB can compensate for due to its non-invasiveness, ease of operation, and real-time continuous monitoring capability. Our future research will focus on a head-to-head comparison of cardiac function assessments derived from TEE and hemodynamic parameters monitored by TEB to further validate the complementary value of the two techniques.

To obtain clinically credible agreement results, we adopted a standardized two-step Bland–Altman analytical framework tailored to repeated intraoperative measurements. Different from the conventional method which merely calculates the percentage of data within 95% LOA, we predefined clinically acceptable LOA thresholds for all indicators prior to data analysis, rather than making subjective post-hoc judgements. Moreover, we corrected the model to eliminate within-patient clustering effects arising from multiple repeated observations per subject, which conventional Bland–Altman approaches would ignore. The corrected 95% LOA in our analysis fully eliminated statistical confounding caused by repeated measures, and the narrow, stable limits confirmed the robustness of the good agreement between TEB and the Swan-Ganz catheter. This design ensures our conclusion has actual clinical meaning, instead of merely satisfying statistical significance.

In addition to volume status, cardiac functional status is the primary determinant of perioperative hemodynamic stability in OPCABG patients. Intuitive and continuous monitoring of CO, SV, and other cardiac function-related indices is therefore of great clinical significance for anesthesiologists to make timely and accurate therapeutic decisions. Previous clinical studies on post-cardiac surgery patients have reported good agreement between TEB and pulmonary artery thermodilution (the gold standard) in the measurement of CO and cardiac index [31,32], which is consistent with the findings of the present study – TEB-derived CO, SV, and SI showed high agreement with those measured by the Swan-Ganz catheter. This concordant clinical evidence may be attributed to recent technological advancements in non-invasive hemodynamic monitors, including optimized impedance signal acquisition algorithms and noise reduction technology, which have significantly improved the reliability and stability of TEB measurements. Sageman et al. also confirmed the non-inferiority of TEB to invasive thermodilution for CO monitoring *via* a rigorous equivalence analysis, further supporting our study’s conclusions [33].

A major clinical concern regarding the intraoperative application of TEB is signal interference, with electrocautery and ECG monitor respiratory function signals being the most common confounding factors [34,35]. In this study, we implemented targeted interference reduction measures: the respiratory monitoring function of the ECG monitor was disabled at the start of TEB monitoring, and data collection was performed during electrocautery-free periods and at hemodynamically stable time points. These standardized procedures effectively minimized signal interference and ensured the reliability of TEB measurements. Despite these interventions, potential confounding factors remained: small amounts of residual intrathoracic gas or blood after OPCABG may alter thoracic tissue impedance, and the placement of the Swan-Ganz catheter itself may cause minor changes in thoracic anatomical structure – both of which could slightly affect TEB-derived parameter values. These factors may partially explain the numerical differences in EDV measurements observed between the two methods and highlight the need for further optimization of TEB technology for intraoperative use in cardiothoracic surgery.

Notably, all patients in this study underwent Swan-Ganz catheter placement, which may have introduced a minor confounding effect on thoracic impedance and TEB measurements, particularly for EDV. However, TEB data were collected in a strictly standardized manner for all patients, and the concordant dynamic trends of all measured parameters confirmed the reliability of TEB for monitoring hemodynamic changes – rather than absolute values – in OPCABG patients. The present study demonstrated that while there were numerical differences in individual parameters (e.g. EDV) between TEB and the invasive gold standard, TEB exhibited good agreement in measuring the key hemodynamic indices (CO, SV, SI) and reliable dynamic trend monitoring for all indices. Notably, EDV had marked mean bias and broad LOA, which precludes TEB from providing clinically acceptable absolute EDV values for direct substitution of Swan-Ganz results; the consistent temporal trend of EDV only supports TEB for serial trend surveillance of preload variation instead of absolute volume measurement. This finding is of great clinical significance, as trend monitoring is more clinically valuable than absolute value measurement for perioperative hemodynamic management in OPCABG patients, where timely identification of hemodynamic changes is critical for guiding treatment.

## 5. Conclusion

This prospective study confirmed that TEB shows satisfactory agreement with Swan-Ganz thermodilution for the absolute values of CO, SV and SI, and can accurately reflect the dynamic perioperative trends of all four hemodynamic parameters in patients undergoing OPCABG. Nevertheless, TEB presents obvious measurement bias for EDV and cannot provide reliable absolute EDV quantification, even though its trend-tracking performance remains valid.

As a non-invasive monitoring modality, TEB features simple operation, low cost and real-time continuous measurement without invasive complications, making it an ideal supplementary trend-monitoring tool under standardized perioperative conditions. Its impedance signal is prone to intraoperative interference from electrocautery and intrathoracic anatomical changes, which accounts for its limited accuracy for EDV measurement compared with the invasive gold standard.

Several limitations of this single-center study should be noted: the sample size is relatively small with no formal a priori power calculation for agreement analysis, only two discrete time points were sampled, and TEE and surgical manipulations inevitably created signal interference. Besides, we excluded severely hemodynamically unstable patients who required emergent conversion to on-pump surgery, so our findings are only applicable to stable OPCABG patients and cannot be generalized to high-risk, critically unstable populations.

In conclusion, for hemodynamically stable patients receiving OPCABG, TEB is capable of accurate absolute measurement of CO, SV and SI as well as reliable trend monitoring for all hemodynamic indices. Clinically, TEB is recommended as a valuable non-invasive supplementary monitoring tool, rather than a complete substitute for invasive Swan-Ganz catheterization.

## Consent to participate

All participants involved in the study signed informed consent forms, clearly indicating their voluntary participation.

## Data Availability

The data supporting the results of this study are available from the corresponding author upon reasonable request, consistent with the Taylor & Francis Share Upon Reasonable Request data policy.
